# Transitioning towards senior medical resident: identification of the required competencies using consensus methodology

**Published:** 2018-07-27

**Authors:** Roy Khalife, Carol Gonsalves, Catherine Code, Samantha Halman

**Affiliations:** 1 Department of Medicine, University of Ottawa, Ontario, Canada; 2 Division of Hematology, University of Ottawa, Ontario, Canada

## Abstract

**Background:**

Residency programs are facing significant restructuring through the “Competence by Design” (CBD) framework proposed by the Royal College of Physicians and Surgeons of Canada (RCPSC). Our goal was to establish the competencies to be acquired during the transition to a senior role within Internal Medicine (IM) training.

**Methods:**

Using a modified Delphi technique, practicing IM physicians and recent graduates were polled to develop consensus on the required competencies to effectively transition from junior to senior medical resident. Participants rated each competency on a three-point Likert scale. Each competency was linked to an Entrustable Professional Activity (EPA) identified by the RCPSC IM Specialty Committee.

**Results:**

A total of eighteen participants took part in item generation (16% response rate) and nineteen in the initial ranking with seventeen completing all three iterations (89% completion rate). Eighty-three competencies were identified during questionnaire development. A final list of seventy-seven competencies reached consensus after three rounds. Most competencies matched to core of discipline EPAs.

**Conclusion:**

This consensus-based list of competencies will help create a framework and tools for the assessment of junior residents as they prepare to transition to the role of senior in the new CBD curricula for IM trainees at our institution.

## Introduction

The continuum of medical education is punctuated by significant transition points. Traditionally, educators and curriculum developers have conceptualized medical training as moving sequentially from the admission process through various stages to finally becoming a “real” doctor. In a white paper prepared for the Royal College of Physicians and Surgeons of Canada (RCPSC) Future of Medical Education in Canada (FMEC) project, four distinct periods of transition are highlighted: entry into medical school, pre-clinical to clinical, undergraduate to postgraduate (residency), and residency to practice.^1^ Each of these transition periods is associated with growing autonomy with the concomitant increase in demands and responsibilities. There is a large body of evidence suggesting that there is often a lack of structured support and standardization across organizations (institutions, programs) to guide individuals through transitions.^1^ As a result, there is emerging interest within the medical education community to better facilitate these transitions and improve the acquisition of competencies throughout the learning continuum.^2^

The Future of Medical Education in Canada Postgraduate (FMEC PG) project, launched in 2010, offered a total of 10 recommendations to best enhance postgraduate medical education.^3^ Recommendation #5 targets transitions period with a focus on transition-to-residency and transition-to-practice.^3^ Many medical schools have looked into the transition to postgraduate training, but these initiatives remain program-specific and not widely adopted.^2^

While much of the focus in the area of transitions has highlighted these traditional passages through medical training, trainees must navigate a series of more nuanced yet crucial steps along the way. To date, there is very little practical evidence available on role of transitions *within* residency training, let alone transitions specific to Internal Medicine (IM) programs. This will soon need reassessment with the staged implementation of competency-based medical education (CBME) across the country. The recently proposed “Competence by Design” (CBD) Competence Continuum by the RCPSC does highlight a key step where junior residents move from “Foundations of Discipline” (FD; broad-based competencies that every trainee must acquire prior to advancing to discipline-specific competencies) to “Core of Discipline” (CD; the specific competencies that make up the majority of the discipline).^4^ There has been a call from the RCPSC to identify and standardize the expectations for transitional periods *within* residency programs taking into account the CBD framework.^1^ Individual disciplines are being charged with defining what competencies are to be included within these transition periods. In IM, the transition from FD to CD is akin to the transition from what we call a junior medical resident (JMR) to senior medical resident (SMR). Although the timing of this transition varies by training program, this commonly occurs after one (such as in our institution) or two years of training. As pointed out by Aschenbrener *et al*., a set calendar date is not truly reflective of trainees’ readiness for this transition.^2^ Within CBD, this process will require deliberate assessment of the competence of JMRs in order to proceed to the next level in their training.

What skills do we expect of our SMRs that we should ensure JMRs obtain in order to be successful? In reviewing the literature, it became apparent that while some studies have addressed gaps in training at transition points,^5-8^ we did not find any study that explicitly identified all the competencies required for the transition from JMR to SMR. In the era of CBME, it is increasingly important that we have clear expectations of the outcomes of training at critical transition points to ensure maximal curricular effectiveness. IM programs will need to restructure their assessment program to fulfill CBD framework requirement from the RCPSC. This involves specifically defining expectations of the competencies to be achieved at each transition stage within the training program.

The purpose of this study was to develop a comprehensive list of the competencies to be acquired by JMRs to facilitate the transition to SMR.

## Methods

This study underwent delegated review and approval was granted by the Ottawa Health Science Network Research Ethics Board (OHSN-REB).

### Study Design

The purpose of the study was to develop a comprehensive list of the required competencies to transition from JMR to SMR. Given that there is no literature that currently provides such as list, we used consensus methodology. In order to survey a large number of individuals asynchronously, we selected a modified Delphi process. The Delphi technique is an iterative consultation of experts without direct interaction giving equal weighting to all individual opinions.^9-10^ It is one of the most commonly used consensus methods in medical education research.^11^ The process of generating the items (questionnaire development) and the Delphi method are described in detail below.

#### Participants

Participants included a sample of practicing IM physicians at a single academic institution and recent graduates from the IM program at the same institution. Typically, participants are “experts” and are knowledgeable in the area of research, representative of the area in question and have practical experience.^11^ In a CBD framework, knowledgeable experts might include program directors, specialty group members and educationalists. We purposefully chose to not sample this population directly and favoured participants with more *practical* experience (end-users). This differs from other studies using similar methodology^12-14^ and brings a different perspective to this field of research. We chose to use a single institution to survey our landscape locally with a plan to pursue national research in the future.

We used a convenience sampling strategy. All IM subspecialty training residents (post-graduate year four and above) within the Department of Medicine at the University of Ottawa and attending physicians within the Division of General Internal Medicine (GIM) at the Ottawa Hospital were invited to participate in the study. Subspecialty residents were recruited as they would have recently completed their core IM training and were identified as additional key stakeholders in providing information regarding competencies they would have needed to transition from JMR to SMR. Attending physicians in the division of GIM needed to specify that they had supervised both JMR and SMR within the last 12 months to be included in the study.

For the questionnaire development, the principal investigator, who was a member of the division, distributed a recruitment email to attending physicians. To minimize any coercion, the recruitment email was distributed by the postgraduate medical education office to participating trainees as the principal investigator is involved in trainee supervision and assessment. The same mode of recruitment applied to the first round of the Delphi process. Participants need not have participated in the questionnaire development to participate in the ranking. For rounds 2 and 3, only participants who completed round 1 were permitted to participate. Participants provided their email address, which was only accessible to the research assistant who sent out the subsequent emails. Participants who completed all three rankings were eligible for a draw for a $200 gift certificate to a local restaurant.

#### Questionnaire development

In a classic Delphi, the questionnaire is developed by the research team.^11^ Our research team includes two medical educators (SH, CG) and two program directors who sit on a RCPSC CBD subspecialty committee (SH, CC). In an effort not to limit item generation and to capture the opinions of end-users, we chose to develop the questionnaire by surveying our participants rather than relying on the research team’s expertise. Participants received an email invitation with a link to the online survey (FluidSurveys^®^). The link was active for a two-week period and participants received a reminder after one week. The survey included an introduction outlining the RCPSC definitions for CBME, competencies and milestones.^15^ The survey was divided into seven sections, each labelled with a specific CanMEDS 2015 role (Medical Expert, Communicator, Collaborator, Leader, Health Advocate, Scholar, and Professional) along with its definition and two examples of competencies that may fit within this role to help guide participants. The examples were suggested by study investigators based on a review of the RCPSC objectives of training specific to IM.^16^ Participants were asked to add as many competencies they felt were pertinent to the JMR to SMR transition within each CanMEDS 2015 role.

Three study investigators reviewed the resulting list of competencies to ensure uniformity of wording and deletion of duplicated items. We discarded items that were deemed non-specific to IM training by at least two investigators. We combined competencies into one list for each CanMEDS role, identified as provided by Resident (R), Attending Physicians (A), or both (R+A).

#### Modified Delphi Process

We carried out the Delphi process in three rounds as determined *a priori*. The recommended number of rounds is typically two to three which may help decrease attrition or participant dropout with successive rounds.^11,17^ In the first round, participants were presented with the list of competencies from the questionnaire development phase. As with the prior phase, a one-page introduction reminding participants of key definitions was provided. Participants were asked to rate each item on a three-point Likert scale: not required, neutral, required. Consensus was defined as a minimum of 75% agreement that a competency was “required” or “not required.” Participants could not add new competencies.

Only items that did not reach consensus were incorporated in the subsequent rounds. The follow-up ranking survey was only addressed to those that had completed the prior ranking. We provided participants with the percentage of responses from each cohort (residents and attending physicians) for each scale category from the prior round for each item. Participants were asked to re-rank items on the same three-point Likert scale. For each round, the survey remained open for two weeks with a single email reminder at the midway point.

### Data Analysis

The list of competencies generated during questionnaire development was submitted for ranking (first round Delphi). Consensus was defined at 75% agreement. Items that reached 75% agreement for “required” were included in the final list. Items that reached 75% for “not required” were discarded. Items that did not reach consensus were resubmitted for ranking through rounds 2 and 3. Items were discarded if consensus on “required” was not reached after three consecutive ranking iterations.

### Data Mapping

As a means of quality control, we attempted to link each final item meeting consensus to one Entrustable Professional Activity (EPA) developed by the RCPSC IM Specialty Committee. Each EPA is described under one of four transition points (transition to discipline, foundation of discipline, core of discipline and transition to practice). It was felt that if competencies were truly applicable to transition from JMR to SMR, most should map to “core of discipline” EPAs. Two study investigators independently mapped each competency to EPAs. When the competency fell within more than one EPA, investigators independently decided which was more appropriate. We calculated initial agreement using Cohen’s kappa. Any disagreement was resolved with discussion between the two investigators.

## Results

### Questionnaire Development

A total of eighteen participants engaged with this phase of the study. Twelve were attending physicians within the division of GIM (out of potential 32) and six (out of potential 84) were subspecialty residents in the Department of Medicine. Combined response rate was low at 16% although better for attending physicians (38%) than residents (7%). A total of 332 competencies distributed across all CanMEDS roles were generated. After removing duplicates, 187 competencies remained.

Table 1 outlines the descriptive statistics of the original list. Overall, a larger number of competencies were suggested by attending physicians with them contributing (either alone or in combination with residents) to 87% of competencies. Residents suggested fewer competencies, with them contributing (either alone or in combination) to 41% of listed competencies. This was consistent across each of the CanMEDS roles.

**Table 1:** Unique competencies generated during questionnaire development and the final number included for ranking after collapse of redundant items and triage of non-specific items

CanMEDS role	Items provided by attending physicians only (%)	Items provided by residents only (%)	Items provided by both participants (%)	Final number of items included in the first iteration after collapse and triage (%)
Medical Expert	23 (59)	4 (10)	12 (31)	20 (24)
Communication	23 (62)	7 (19)	7 (19)	14 (17)
Collaborator	8 (44)	3 (17)	7 (39)	14 (17)
Leader	17 (61)	5 (18)	6 (21)	10 (12)
Health Advocate	22 (73)	3 (10)	5 (17)	9 (11)
Scholar	11 (55)	2 (10)	7 (35)	10 (12)
Professional	6 (40)	1 (7)	8 (53)	6 (7)
Total	110 (59)	25 (13)	52 (28)	83 (100)

We reviewed the list for items that we felt could be collapsed or for items that we felt were not specific to IM. An example of an item that we felt was not specific to IM was the “ability to interpret laboratory investigations.” An example of two items that we collapsed includes the “ability to advocate for health issues for refugees” which was collapsed into the more inclusive “ability to advocate for health issues for marginalized populations.” Many competencies were listed under more than one CanMEDS role. We removed and classified these items under the most appropriate role based on agreement from at least two of three study investigators who are all very familiar with CanMEDS. As outlined in Table 1, a total of 83 competencies were left at the end of this process and included in the first round of the Delphi.

### First round Delphi

We recruited from the initial larger pool of participants for the first round in an effort to improve response rate. A total of 19 participants took part in round one, including nine attending physicians (response rate = 28%) and 10 subspecialty residents (response rate = 12%). Participants knew whether the proposed competency was suggested by attending physicians only (A), residents only (R) or both (A+R) as well as response frequency.

### Second and third round Delphi

Any competency that did not reach consensus by any of the three groupings (A, R or A+R) was included in the second round. The same process was repeated for round three with the exception that we grouped all participants into one pool rather than having different links for residents and attending physicians. Participants were still presented with the frequency for each item being re-ranked but these were not separated into the three groupings above. The original need for two separate links for recruitment purposes had to do with potential coercion if the link was distributed by one of the primary investigators. Given that only participants who completed the first round were subsequently contacted via the research assistant and that no identifying data were collected (included whether they were attending physicians or residents), this was not possible for further iterations. Participant dropout rate was low with 89% of participants completing all three rounds (n=17). Table 2 outlines the number of competencies that reached consensus with every round. A total of 77 competencies were included in the final list and six were discarded after not achieving consensus upon completing three rounds of ranking. The final list of competencies in provided in Appendix A.

**Table 2:** Number of competencies that reached consensus after each round within each of the CanMEDS role

CanMEDS Role	Number of original competencies	Number of consensus after 1^st^ ranking (%)	Number of consensus after 2^nd^ ranking (%)	Number of consensus after 3^rd^ ranking (%)	Total number that reached consensus (%)	Number of discarded competencies (%)
Medical Expert	20	10 (50)	5 (25)	1 (5)	16 (80)	4 (20)
Communicator	14	9 (64)	3 (21)	0	12 (86)	2 (14)
Collaborator	14	13 (93)	1 (7)	0	14 (100)	0
Leader	10	6 (60)	2 (20)	2 (20)	10 (100)	0
Health-Advocate	9	3 (33)	3 (33)	3 (33)	9 (100)	0
Scholar	10	7 (70)	2 (20)	1 (10)	10 (100)	0
Professional	6	3 (50)	1 (17)	2 (33)	6 (100)	0
Total	83	51 (61)	17 (20)	9 (11)	77 (93)	6 (7)

### Data linking to EPAs

In an effort to ensure that these competencies were aligned with the Competence by Design framework, two study investigators (SH & RK) independently linked each of the 77 competencies to the 29 EPAs developed by the RCPSC IM CBD working group. The language of the EPAs is still under development at the RCPSC and cannot be shared at this time. Permission to use the current EPAs was granted by the RCPSC given that one co-author for this study (CC) sits on that working group. Overall inter-rater reliability was moderate (κ 0.63, 95% CI: 0.52-0.74).^18^ Inter-rater reliability was very strong for competencies within the medical expert (κ 0.79) and communicator roles (κ 1.00) whereas there was no initial rater agreement within the health advocate and professional roles (κ 0). After discussion between raters, none of the competencies mapped to “transition to discipline,” 18 (23%) mapped to “foundation of discipline,” 41 (53%) to “core of discipline,” and 11 (14%) to “transition to practice.” We were unable to map seven (9%) competencies to any EPA. The mapping of competencies to EPAs is available in Appendix A.

## Discussion

The Delphi technique has been used for curriculum development at the various stages of medical education.^19-21^ We chose a modified Delphi process for this study because it allows consensus building amongst potentially large groups of participants without direct interaction and it is easily adaptable to be done electronically. This study used a modified Delphi process to specifically identify competencies required at the transition point between junior and senior medical residents in IM training.

It is not surprising that a large proportion of the original competencies were suggested under the role of “medical expert” (24%). Comparable results have been shown in similar studies and likely reflect current models of medical education where knowledge and skills are seen as crucial building blocks by clinical educators.^19^ Where medical knowledge is fairly easy to assess with our current assessment methods, program directors have reported concerns about how the other roles are taught and evaluated in their programs.^22-24^ We were therefore not surprised to find that fewer competencies were included for some “non-medical expert” roles such as health advocate and professional.^23-24^ Appreciating and defining these types of roles may be more challenging to our participants as physicians don’t necessarily have specific expertise in these areas compared to medical knowledge which is a common denominator.

Six of the original 83 items we proposed for ranking during the Delphi rounds did not reach consensus. Of these items, four were in the medical expert role (perform appropriate perioperative consultation, manage pregnancy-related medical conditions, demonstrate basic point-of-care ultrasound (POCUS) knowledge, and recognize and manage atypical disease presentations) and two were in the communicator role (explain the complex/hierarchal structure of care within the hospital and counsel patients about limitations and roles of alternative medical therapies).

A review of the objectives of training in IM reveals that there are indeed objectives specific to the care of the pregnant patient and the perioperative assessment and management of patients with specific conditions.^16^ That said, it is likely that these two items did not reach consensus as these areas are now felt to be closer aligned to the objectives of training in the subspecialty of GIM. After many years of seeking recognition as a distinct field, GIM was officially accepted as a unique subspecialty of the RCPSC in 2010. In the definition of a general internist, it is explicitly stated that these physicians are prepared to maintain the stability of patients “during physiological stresses such as during pregnancy or the peri-operative period.”^25^ We suspect that attending physicians in the division of GIM were attuned to this distinction in scope of practice and thus these items did not reach consensus for IM trainees. Conversely, the use of POCUS has increased substantially in the last decade with Emergency Medicine and Critical Care leading the way.^26^ The is a growing body of evidence that POCUS has a role to play in IM but until recently, there was little consensus on what the curriculum should include.^26,27^ This year, recommendations regarding curricular content were made by the Canadian Internal Medicine Ultrasound (CIMUS) Group.^28^ We suspect that these recommendations will lead to a broader use of POCUS in IM and will likely result in this competency being adopted in the near future. Finally, within the medical expert role, the last competency to not reach consensus for inclusion related to “atypical disease presentations.” Items that did reach consensus for inclusion described the care of “complex” and “undifferentiated” patients which likely better captured what we expect our JMRs to achieve rather than “atypical” presentations which often elude even the most senior clinicians.

The inter-rater reliability when linking 77 competencies to 29 possible EPAs was moderate (κ 0.63). This is in part because one rater initially felt that several items could not be linked to EPAs. At the end of the exercise, we still could not map one competency in health advocate and all six of the professional competencies to EPAs. When these 7 competencies are excluded, Cohen’s kappa improves to κ 0.69 (95% CI 0.58-0.80). It is possible that the health advocate and professional roles are currently underrepresented in the IM EPAs and this will require some attention. As expected, most of our competencies were mapped to the “core of discipline” stage of residency. Within the CBD framework, this is where we would expect SMRs would fall. Although we did not initially set out to link competencies with EPAs, this adds strength to our study by demonstrating some alignment with the national opinion given that the RCPSC Specialty Committee includes all of the IM program directors from across the country. Of note, our competencies are not meant to replace EPAs but rather fall under them akin to milestones.

Speciality working groups across the country are developing their EPA and milestone lists. Current practices rely very heavily on program directors. We suggest that using consensus methodologies with broader stakeholders may help lessen the burden on program directors to identify some of the competencies or milestones that may help further define EPAs along with providing valuable insights from trainees that may be otherwise overlooked.

### Study limitations

Our response rate was much lower than projected, especially from the resident cohort. This is a recognized challenge of a multi-step process such as the Delphi. Many factors may have contributed including the timing of the study closer to the certification examination for part of the resident cohort (thus making them less likely to participate) and the small number of reminders that was sent out to avoid overburdening participants. It is possible that residents felt this study would not reap any immediate benefits given that they had already completed this step in their training.

Numbers of participants in Delphi studies have ranged from 10 to over 1000.^29^ Whilst we aimed for a higher response rates, the resulting groups of 18 (questionnaire development) and 19 (ranking) participants are sufficient to be considered an appropriate size.^30^ Our drop-out rate was very low with almost 90% of participants completing all ranking iterations.

We acknowledge that our resulting list of competencies is large with 77 items remaining after the final ranking iteration. Although this represents a small reduction from the original list (7% reduction), participants were provided with the status of the group’s collective opinion with each round and were provided with the option to revise their opinion based on the forming group opinion.

Recruitment of attending physicians was limited to the division of GIM as the bulk of the training and residents’ assessment is completed within this division. However, contribution from other medical subspecialties involved in the education of IM trainees could be useful and improve data collection. This was a single centre study. Future work is needed to determine the generalizability of the data and the feasibility of this methodology in other settings.

### Conclusion

Using consensus methodology through a rigorous modified Delphi process, this study identified competencies required at the transition period between junior and senior residents in IM training. This study advances work in the area of transitions by focusing on a critical period *within* residency training. It may also serve as a template for further study in other disciplines charged with identifying such transitions within their own training programs in the era of CBME.

## Appendix A

List of competencies mapped to CanMEDS roles and EPAs.

The following abbreviations have been used in the tables below: transition to discipline (TD), foundation of discipline (FD), core of discipline (CD), and transition to practice (TP).

### MEDICAL EXPERT

**Table:**

1.	Elicit an appropriate and prioritized differential diagnosis	CD1
2.	Perform independently Internal Medicine specific procedures at the patient’s bedside (Lumbar Puncture, Knee Arthrocentesis, Central Line, Intubation, Paracentesis, Thoracentesis)	CD5
3.	Interpret basic diagnostic imaging studies (e.g. CXR, CT Head, etc.)	FD1
4.	Interpret Electrocardiograms	FD1
5.	Demonstrate the medical knowledge to manage complex medical illnesses	CD1
6.	Demonstrate appropriate knowledge, approach and skills for the management of undifferentiated medical problems	TP3
7.	Demonstrate the ability to diagnose dementia and frailty and be able to include these as prognostic factors in decision making about interventions	CD7
8.	Recognize & manage critically/acutely ill patient, including the need for resuscitation measures & ICU.	CD4
9.	Recognize and appropriately respond to changes in patients’ medical condition/stability	FD2
10.	Triage and prioritize patients based on their illness, its severity, and their clinical status	FD1
11.	Demonstrate the ability to effectively triage medical consultation requests (ER, Inpatient services)	CD3
12.	Manage and lead a cardiac arrest situation	CD4
13.	Demonstrate an understanding of the risks of polypharmacy and be able to prescribe effectively	CD10
14.	Demonstrate adequate knowledge regarding prescribed medication(s) including interactions and side-effects	CD10
15.	Apply clinical guidelines to patient care	FD7
16.	Organize an appropriate discharge plan for a patient	FD4

### COMMUNICATOR

**Table:**

1.	Communicate verbally in concise, clear and empathetic fashion	CD7
2.	Engage and communicate with ‘difficult’ patients and/or families	CD7
3.	Provide explanation on diagnosis, test results, therapies and prognosis to patient and/or families using non-medical terminology	CD7
4.	Engage & connect the medical team with patients, their families and POA	CD7
5.	Update families on their loved ones	CD7
6.	Facilitate and lead a family meeting	CD7
7.	Ability to disclose adverse event/medical errors to patients and/or families	CD8
8.	Discuss goals of care and resuscitation measures with patients and/or families	FD6
9.	Discuss and address end-of-life related issues	CD9
10.	Demonstrate the ability to break bad news	CD7
11.	Seek to obtain informed consent	CD6
12.	Demonstrate the ability to assess patient’s competency	CD6

### COLLABORATOR

**Table:**

1.	Demonstrate the ability to consult other medical services appropriately	FD3
2.	Demonstrate the ability to consult allied health professionals appropriately	FD3
3.	Work effectively within a multi-disciplinary medical team	TP6
4.	Participate and conduct an effective family meeting in collaboration with other health care professionals	CD7
5.	Demonstrate the ability to respond to nursing concerns	TP6
6.	Provide effective handover for continuity of care	TP5
7.	Communicate with primary care physicians and offer update about mutual patient	TP6
8.	Establish clear and concise written and verbal management plans for patients with complex and multiple medical issues	CD1
9.	Provide timely and effective consults when requested	CD3
10.	Demonstrate the ability to dictate comprehensive and focused consultation notes	CD3
11.	Demonstrate the ability to dictate comprehensive admission notes	FD1
12.	Demonstrate the ability to dictate comprehensive discharge plans	FD4
13.	Demonstrate the ability to review with junior trainees	CD11
14.	Demonstrate the ability to communicate with ER to gain all the information regarding an incoming consult	CD3

### LEADER

**Table:**

1.	Demonstrate leadership in the management of a medical in-patient team overnight	CD11
2.	Demonstrate leadership in the management of a medical in-patient team during routine day-to-day activities	TP1
3.	Demonstrate the ability to lead bedside rounds	CD11
4.	Demonstrate the ability to lead structured teaching activities	CD11
5.	Demonstrate the ability to provide guidance and teaching opportunities to junior trainees	CD11
6.	Demonstrate the ability to identify and assist other trainees who are struggling to fulfill their duties within the team	CD11
7.	Demonstrate mentorship and role model qualities	FD7
8.	Demonstrate commitment to a culture of wellness	FD7
9.	Demonstrate the ability to step in to handle emergencies as needed	CD4
10.	Allocate and use limited health-care resources wisely	TP8

### HEALTH-ADVOCATE

**Table:**

1.	Demonstrate understanding of an involvement in patient safety/quality improvement initiatives	TP8
2.	Recognize patients requiring community support and services, and mobilize those resources	CD10
3.	Demonstrate an understanding of the health care barriers based on the determinants of health related to the population served	TP8
4.	Mobilize resources and provide adequate continuity of care for marginalized populations	CD10
5.	Advocate and recognize health issues specific to various ethnic groups	CD10
6.	Recognize and manage elder abuse	N/A
7.	Recognize and manage substance abuse disorders	CD10
8.	Counsel and promote healthy lifestyle changes	CD10
9.	Assess & discuss importance of medication adherence	CD7

### SCHOLAR

**Table:**

1.	Demonstrate commitment for self-learning	TP7
2.	Demonstrate the ability to identify one’s knowledge gaps and learning needs	FD7
3.	Demonstrate an effective presentation at Journal Club, Rounds, Morning report and/or conferences.	TP7
4.	Demonstrate the ability to search the medical literature for accurate & up-to-date information, best practice measures, guidelines, etc.	FD7
5.	Demonstrate the ability for critical appraisal of the medical literature	FD7
6.	Integrate evidence-based medicine in everyday care	FD7
7.	Teach clinical skills to junior trainees	CD11
8.	Teach procedural skills to junior trainees	CD11
9.	Participate in the evaluation of junior trainees	CD11
10.	Organize a learning plan for the medical team	FD7

### PROFESSIONAL

**Table:**

1.	Demonstrate the ability to apply the principles and limits of patient confidentiality	N/A
2.	Demonstrate reliability & commitment to complete tasks & duties	N/A
3.	Demonstrate a commitment to maintain a work-life balance	N/A
4.	Recognize and takes appropriate action to deal with one’s limits	N/A
5.	Manage conflicts within the workplace	N/A
6.	Manage unprofessional behaviors in the workplace	N/A
